# Network Protein Interaction in Parkinson’s Disease and Periodontitis Interplay: A Preliminary Bioinformatic Analysis

**DOI:** 10.3390/genes11111385

**Published:** 2020-11-23

**Authors:** João Botelho, Paulo Mascarenhas, José João Mendes, Vanessa Machado

**Affiliations:** 1 Periodontology Department, Clinical Research Unit (CRU), Centro de Investigação Interdisciplinar Egas Moniz (CiiEM), Instituto Universitário Egas Moniz (IUEM), 2829-511 Caparica, Portugal; vmachado@egasmoniz.edu.pt; 2 Evidence-Based Hub, Clinical Research Unit (CRU), Centro de Investigação Interdisciplinar Egas Moniz (CiiEM), Instituto Universitário Egas Moniz (IUEM), 2829-511 Caparica, Portugal; pmascarenhas@egasmoniz.edu.pt (P.M.); jmendes@egasmoniz.edu.pt (J.J.M.); 3 Center for Medical Genetics and Pediatric Nutrition Egas Moniz, Instituto Universitário Egas Moniz (IUEM), 2829-511 Caparica, Portugal

**Keywords:** Parkinson’s disease, periodontitis, periodontal disease, protein–protein network interaction, bioinformatics

## Abstract

Recent studies supported a clinical association between Parkinson’s disease (PD) and periodontitis. Hence, investigating possible interactions between proteins associated to these two conditions is of interest. In this study, we conducted a protein–protein network interaction analysis with recognized genes encoding proteins with variants strongly associated with PD and periodontitis. Genes of interest were collected via the Genome-Wide Association Studies (GWAS) database. Then, we conducted a protein interaction analysis, using the Search Tool for the Retrieval of Interacting Genes/Proteins (STRING) database, with a highest confidence cutoff of 0.9 and sensitivity analysis with confidence cutoff of 0.7. Our protein network casts a comprehensive analysis of potential protein–protein interactions between PD and periodontitis. This analysis may underpin valuable information for new candidate molecular mechanisms between PD and periodontitis and may serve new potential targets for research purposes. These results should be carefully interpreted, giving the limitations of this approach.

## 1. Introduction

Parkinson’s disease (PD) is the second most frequent neurodegenerative condition, affecting primarily the central nervous system [1]. PD is clinically characterized by motor and non-motor symptoms, though its clinical onset and progression differ [2], and ultimately lead to disability and poor quality of life [3]. PD is age-dependent and is more prevalent in men [4,5]. The cause of PD is still unknown, however, a recent Mendelian randomization research reported 12 exposures and risk of PD [6]. Further, the role of inflammation in PD has been widely investigated [7,8].

Periodontitis is a chronic dysbiotic and inflammatory disease of the periodontium and one of the most prevalent diseases worldwide [9,10]. This condition presents inflamed gum and alveolar bone loss surrounding the teeth and may cause their loss [11]. Periodontitis has been highly associated with several systemic conditions, for instance, diabetes [12], cardiovascular diseases [13,14], fertility-related conditions [15,16], rheumatic diseases [17] or Alzheimer’s disease [18,19,20]. In most of these diseases, periodontitis shapes its influence through its chronic inflammatory burden and systemic bacteria spread.

The interplay between PD and periodontitis is still scarce, but a number of studies have revealed that the associated motor impairments and cognitive decline may hamper oral hygiene and deteriorate oral health [21,22]. Moreover, PD individuals seem to be at high risk of developing periodontitis [23,24,25,26,27], and this may lead to systemic leukocytosis [28]. Moreover, a nationwide study concluded that people with periodontitis were at more risk to develop PD [29], and one of the possible reasons may be genetic interactions; thus, investigating such a genetic relation would be of great research interest.

Studying possible biological mechanisms between these two diseases could be fruitful towards unexplored ways, and therefore bioinformatics is an appealing resource. In this sense, open-source genomic databases are important for the development of genetic discoveries and possibly for the implementation of clinical decision-making. For instance, protein–protein interaction (PPI) networks have been used to identify genes that are significant in the context of such associations [30,31,32,33,34,35,36].

To this end, we aimed to develop a PPI network between known genes where single nucleotide polymorphisms (SNPs) produce protein variants strongly associated with PD and periodontitis onset, to identify potential biological mechanisms of interaction. Furthermore, we tested the blood–brain barrier permeability of proteins derived from the developed PPI network, to investigate the possibility of moving into the brain.

## 2. Materials and Methods 

### 2.1. Data Source

We searched The National Human Genome Research Institute–European Bioinformatics Institute Catalog of human Genome-Wide Association Studies (NHGRI–GWAS) for PD and periodontitis associated SNPs [37]. This a comprehensive catalogue of reported associations from published Genome-Wide Association Studies (GWAS). We used a publicly available summary statistics dataset from periodontitis GWAS performed in up to 100,903 individuals of European, Asian, American and other ancestries [38,39,40,41,42,43,44,45,46,47,48,49,50] (Table S1).

For Parkinson’s disease, we used a summary statistics dataset from periodontitis GWAS performed in up to 1,640,901 individuals of European, Asian, American, Sub-Saharan African and other ancestries [51,52,53,54,55,56,57,58,59,60,61,62,63,64,65,66,67,68,69,70,71,72,73,74,75,76,77,78,79,80,81,82,83,84,85,86,87,88] (Table S2). GWAS datasets for both PD and periodontitis were derived from different populations, as there are no GWAS data combining both conditions.

### 2.2. Protein–Protein Interaction Networks Functional Enrichment Analysis

The STRING (Search Tool for the Retrieval of Interacting Genes/Proteins) database, complemented with heuristic methods of association and analysis, was used to investigate known and predicted PPI association for both PD and Periodontitis. The STRING database generates a network of PPI from high-throughput experimental data, literature and predictions based on genomic context analysis [89,90]. The interactions in STRING are sourced from five main sources: Genomic Context Predictions, High-throughput Lab Experiments, (Conserved) Co-Expression, Automated Text mining and Previous Knowledge in Databases. Protein characteristics were obtained through the Universal Protein Resource [91].

### 2.3. Blood–Brain Barrier Permeability Analysis 

Blood–brain barrier permeability was predicted through the protein characteristics presented in the Protein Atlas Database [92]. Protein information (length, mass, prediction as a signal peptide and prediction as transmembrane protein), as well RNA expression within brain tissues, allowed us to foresee the possibility of passing. We assumed that isoforms having a mass equal to or lower than 20 kDa were deemed possible to surpass the blood–brain barrier permeability. Proteins with a mass higher than 20 kDa were assumed to be found at the brain via local expression and secretion. 

### 2.4. Data Management, Test Methods and Analysis

Data were downloaded through the GWAS website and handled with Microsoft Office Excel. PPI network was rendered via STRING database version 10.5. We set the highest confidence cutoff in this interaction analysis (of 0.9). Then, a sensitivity analysis with a confidence cutoff of 0.7 was conducted, to investigate whether the results are dependent on the choice of the confidence cutoff. In the resulting PPI network, proteins are presented as nodes which are connected by lines whose thickness represents this confidence level. We also carried out a heatmap of protein interactions, using the ‘ggplot’ R package.

### 2.5. Protein Set Enrichment Analysis

To investigate the presence of over-representation of some proteins in the obtained network and the possible influence in the observed PPI, we used The Geneontology Resource (http://geneontology.org/). We started by analyzing the “Panther pathways” [93] over the final list of genes in the PPI. Then, we looked over the “Reactome pathways”. To calculate the false discovery rate, both annotations were tested through the Fisher’s Exact test.

## 3. Results

### 3.1. Protein–Protein Interaction Analysis

**Figure 1:**
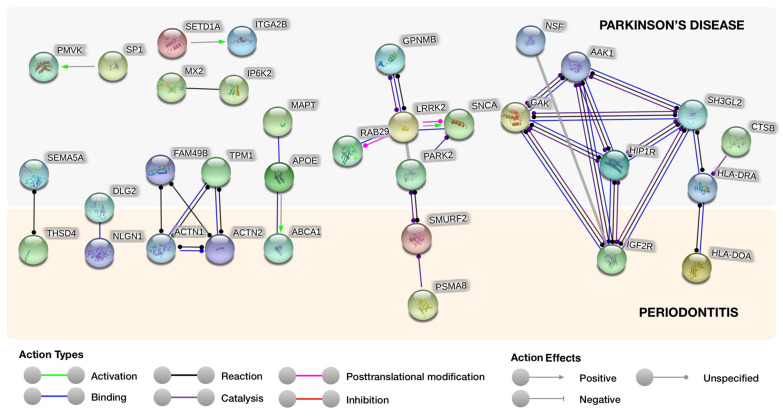
Search Tool for the Retrieval of Interacting Genes/Proteins (STRING) analysis reveals protein interaction networks between Parkinson’s disease and periodontitis proteins. We implemented the highest confidence cutoff of 0.9 in this network. In the resulting protein association network, proteins are presented as nodes which are connected by lines whose thickness represents the confidence level (0.9).

Using the STRING online tool, we found 100 nodes with 66 PPI relationships (Figure 1). The properties of the network were analyzed, indicating that the network of PPIs had more interactions among themselves than what would be expected for a random set of proteins of similar size, drawn from the proteome. Such an enrichment indicates that the proteins are, at least, partially biologically connected (*p*-value = 1.89 × 10^−5^). From an expected number of 14 edges, it was cast a final number of 33 edges (average node degree = 0.66; average local clustering coefficient = 0.27). The sensitivity analysis with a confidence cutoff of 0.7 revealed that the results are not dependent on the choice of the confidence cutoff, since the same network emerged.

**Figure 2:**
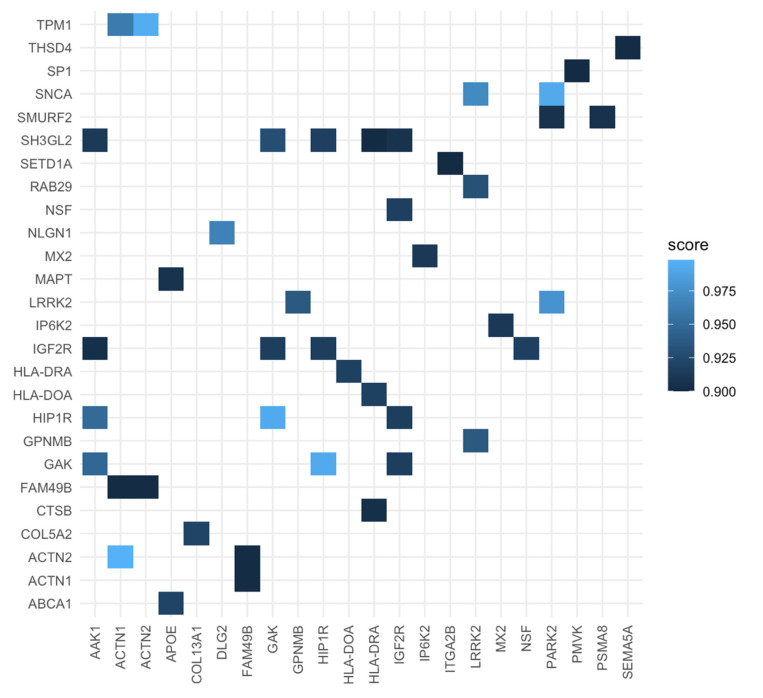
Heatmap of proteins.

Interestingly, we found possible PPIs between PD’s and periodontitis’s known associated proteins (Figure 1 and Table 1). The least likely association is between Disks large homolog 2 (DLG2) and Neuroligin-1 (NLGN1) (Score = 0.966), as DLG2 is a common protein for both conditions. The remaining interactions were as follows: Thrombospondin type 1 domain containing protein 4 (THSD4) and Semaphorin 5A (SEMA5A); Actinin α 1 (ACTN1) and Actinin α 2 (ACTN2) with Family with sequence similarity 49 member B (FAM49B) and Tropomyosin 1 (TPM1); Smad ubiquitination regulatory factor-2 (SMURF2) was establishing a connection between Parkin 2 (PARK2) and Proteasome 20S Subunit α 8 (PSMA8); multiple interactions of insulin-like growth factor 2 receptor (IGF2R) with Huntingtin Interacting Protein 1 Related (HIP1R), Cyclin G-associated kinase (GAK), SF3GL2 and AP2 Associated Kinase 1 (AAK1); HLA class II histocompatibility antigen, DO α chain (HLA-DOA) with HLA class II histocompatibility antigen, and DR α chain (HLA-DRA). Furthermore, we detail the physiological characteristics and localization of each interaction protein (Table 2). We also confirmed protein interaction by using a heatmap plot (Figure 2).

### 3.2. Hydrophobicity Levels of Proteins of Interest

**Figure 3:**
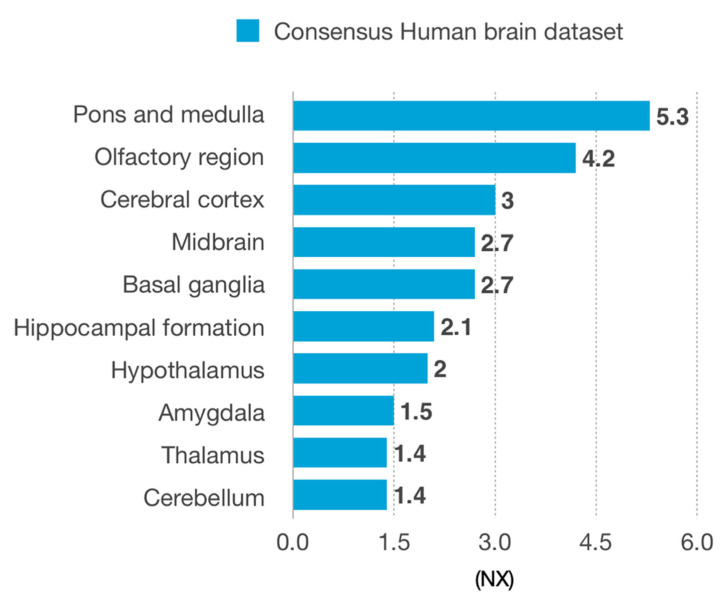
RNA expression of *THSD4* gene in different brain regions, according to the Consensus Human Brain Dataset.

THSD4, as an extracellular matrix protein, was deemed a candidate to pass the blood–brain barrier. Four isoforms were reported: THSD4-201, THSD4-202, THSD4-203 and THSD4-207. The isoforms THSD4-201, THSD4-202 and THSD4-203 have no potential to pass the blood–brain barrier due to a large mass (>20 kDa). A potential candidate is the isoform THSD4-207 (10.7 kDa), which is predicted as a membrane protein. Nevertheless, RNA expression revealed significant expression of THSD4 in several brain areas significantly related with PD, indicating that THSD4 may be produced locally rather than transported into the brain (Figure 3). 

### 3.3. Gene Enrichment Assessment

The Panther over-representation test revealed no statistically significant results. The analysis under the “Reactome pathways” revealed some over-representation in terms of Plasma Lipoprotein Assembly, Golgi Associated Vesicle Biogenesis, trans-Golgi Network Vesicle Budding, Membrane Trafficking, cargo recognition for clathrin-mediated endocytosis, Clathrin-mediated endocytosis, Platelet degranulation and response to elevated platelet cytosolic Ca2+ (Table S3).

## 4. Discussion

In this bioinformatic study, we predicted a potential PPI network between PD and periodontitis from catalogues of human genome-wide association studies, using a bioinformatic approach. Although these PPIs require further experimental validation, they unravel new clues for downstream studies and propose biological-mechanism pathways through which these two conditions may interplay.

A strong candidate in this study is the interaction established by SMURF2, a E3 ubiquitin–protein ligase which accepts ubiquitin from an E2 ubiquitin-conjugating enzyme in the form of a thioester and then directly transfers the ubiquitin to targeted substrates [94]. According to the obtained network, SMURF2 is proposed to interact with PARK2 (E3 ubiquitin–protein ligase parkin), a protein involved in the pathway protein ubiquitination, and previously associated to pathogenic mechanisms in PD [95,96]. Emerging evidence highlighted the role of impaired ubiquitin phosphorylation-dependent mitophagy and PD pathogenesis and supports multiple potential therapeutic targets for PD drug discovery [97,98].

The proteins IGF2R and HLA-DOA also figured some potential role in this network. IGF2R is a transport of phosphorylated lysosomal enzymes from the Golgi complex and the cell surface to lysosomes, and was linked to proteins located at the plasma membrane (AAK1, SH3GH2), perinuclear region (HIP1E, GAK) and others. Moreover, HLA-DOA, a key modulator in the HLA class II restricted antigen presentation pathway, was linked to HLA-DR that binds peptides derived from antigens that access the endocytic route. Interestingly, both IGF2R and HLA-DOA have never been investigated in periodontal medicine, though these potential interactions mainly in the lysocytic/endocytic pathways should be investigated in regard to the interplay between PD and periodontitis.

In the same way, ABCA1, a cAMP-dependent and sulfonylurea-sensitive anion transporter present in the endosome and plasma membrane also depicted a potential link with APOE that mediates the binding, internalization and catabolism of lipoprotein particles. A recent study showed that oxysterols increased the osteogenic activity of PDLSCs, and the expression of ABCA1 increased significantly during osteogenesis [99]. Moreover, APOE—in particular, its isoform 4—was recently proposed to increase the risk of periodontitis [100], and APOE-2 allele is associated with higher prevalence of sporadic PD [101,102]. Thus, the APOE-ABCA1 pathway might play a role in this relationship, mainly within the catabolism of triglycerides and cholesterol, highly associated with PD and periodontitis.

Additionally, THSD4 revealed a possible link with SEMA5A. THSD4 is a protein present in the extracellular region that is weakly expressed in the early stage dental follicle, but becomes readily detectable in assembled microfibril-like structures during the periodontal ligament-forming stage of the dental follicle and in organized microfibrils in the adult periodontal ligament. Moreover, THSD4 is upregulated in the periodontal ligament during periodontitis wound healing (Manabe et al. 2008). On the other hand, SEMA5A is involved in axonal guidance and, in some conditions, reduces the ability to form connections with other neurons in certain brain areas and is possibly a PD preclinical marker [103,104,105]. Considering this possible association, we further analyzed if THSD4 had the ability to pass the blood–brain barrier, though the current knowledge is that its isoforms are too large or are membrane-like proteins; however, the possibility of THSD4 transport proteins in the blood–brain barrier cannot be excluded. Still, a considerable expression of THSD4 is reported in several brain areas, particularly in the basal ganglia and midbrain, known to be PD-related areas. Notwithstanding, medulla/olfactory bulbs are proposed as two starting points of PD in the brain, based on the Braak staging proposal [106], and they accounted for the higher accounts of THSD4 RNA. Hence, despite the fact that the areas more commonly known as PD-related (the midbrain and basal ganglia) present significant values, the reader should bear in mind that this protein of interest is present throughout the brain; all of these regions are affected in PD.

The proteins ACTN1 and ACTN2, both f-actin crosslinking proteins thought to anchor actin to a variety of intracellular structures, were linked with FAM49B and TPM1 present in mitochondria and cytoskeleton.

Furthermore, the protein interaction between HLA-DOA and HLA-DRA may occur through their co-localization at the lysosomal complex; however, the importance for the interaction between these two conditions shall be clarified. The same is expected for the complex IGF2R.

This study presents a powerful and comprehensive analysis from large outputs and large sample size sets. Nevertheless, there are some potential limitations to mention. Firstly, the number of genes represented in GWAS is always dependent on the available number of GWAS studies. Therefore, we anticipate that the increase in GWAS datasets will ultimately unveil new pathways of interaction and disregard previous ones. Another limitation of this study is that we were unable to explore confounding factors, yet the rationale of using GWAS studies is to surpass the environment risk factors load. GWAS research has revolutionized the field of complex disease genetics and has been successful in identifying novel variant–trait associations; this research has limited clinical predictive value [107], but, in our opinion, ignoring these potentially new mechanisms would be unwise. Moreover, the quantity of SNPs of interest in these datasets have combined both European, Asian, African and other populations, which may limit the application of these results. Moreover, the likelihood of finding the number of interactions for each given gene/protein was not possible to clarify, as there are more active genes that may have more interactions, and this should be clarified in future investigations. Despite these limitations, the sample size of this study (over 1.7 million people) makes the results compelling. We have also conducted a protein-enrichment analysis, to look at possible over-representation of influence in the observed PPI.

## 5. Conclusions

Within the limitations of this study, our protein network casts potential protein–protein interactions between Parkinson’s disease and periodontitis. Our results may guide future studies in molecular mechanisms between Parkinson’s disease and periodontitis and may serve new potential targets for research purposes.

## Figures and Tables

**Table 1:** Score results between Parkinson’s disease (PD) and periodontitis related proteins identified in the network interaction.

Proteins for PD (Regulation)	Proteins for Periodontitis (Regulation)	Score
TPM1	ACTN2	0.995
DLG2	NLGN1	0.966
TPM1	ACTN1	0.961
APOE	ABCA1	0.921
HLA-DRA	HLD-DOA	0.918
NSF	IGF2R	0.917
HIP1R	IGF2R	0.916
GAK	IGF2R	0.916
SH3GL2	IGF2R	0.907
PARK2	SMURF2	0.906
AAK1	IGF2R	0.903
SEMA5A	THSD4	0.902
FAM49B	ACTN1	0.901
FAM49B	ACTN2	0.901

**Table 2:** Details of the identified proteins in the interaction between PD and periodontitis.

Protein Symbol	Name	Description	Localization
**Parkinson’s Disease**			
SEMA5A	Semaphorin-5A	Bifunctional axonal guidance signal via sulfated proteoglycans regulation.	- Plasma membrane - Extracellular exosome
FAM49B	Protein FAM49B	Family with sequence similarity 49 member B	- Mitochondrion
TPM1	Tropomyosin α-1 chain	Tropomyosin 1	- Cytoskeleton
APOE	Apolipoprotein E	Intermediates catabolic, link, and internalization processes of lipoprotein particles.	- Extracellular region or secreted
PARK2	E3 ubiquitin–protein ligase parkin	Acts in a multiproteic complex (E3 ubiquitin ligase), catalyzing the link of proteins-ubiquitin moieties. Intervenes monoubiquitination as well as ’Lys-6′, ’Lys-11′, ’Lys-48′-linked and ’Lys-63′-linked polyubiquitination of substrates depending on the context.	- Mitochondrion - Nucleus - Cytosol - Endoplasmic reticulum
HIP1R	Huntingtin-interacting protein 1-related protein	Constituent of vesicles and pits coated by clathrin, that may bind the endocytic apparatus to the actin cytoskeleton. Binds 3-phosphoinositides (through the ENTH domain). May uphold cell survival via stabilization of tyrosine kinases receptor after endocytosis	- Perinuclear - Endomembrane system - Clathrin-coated vesicle membrane
GAK	Cyclin-G-associated kinase	Is a serine/threonine kinase that links with CDK5 and cyclin G acting in the cell cycle and focal adhesion	- Golgi apparatus - Perinuclear region - Focal adhesion
AAK1	AP2-associated protein kinase 1	Regulates endocytosis mediated by clathrin-mediated via AP2M1/mu2 subunit phosphorylation of the adaptor protein complex 2 (AP-2) fostering high affinity binding of AP-2 to cargo membrane proteins during the initial stages of endocytosis.	- Plasma membrane - Clathrin-coated pit - Presynapse
SH3GL2	Endophilin-A1	Involved in the endocytosis of synaptic vesicles.	- Endosome - Cytoplasm - Membrane - Presynapse
CTSB	Cathepsin B	A Thiol protease that is implicated in intracellular degradation and proteins turnover.	- Lysosome - Plasma Membrane - Extracellular region
HLA-DRA	HLA class II histocompatibility antigen, DR α chain	Binds antigens’ peptides that entree into the endocytic path of antigen presenting cells and bestows onto the cell surface for recognition by T-CD4 cells.	- Golgi apparatus - Lysosome - Plasma membrane - Endoplasmic reticulum - Endosome
NSF	Vesicle-fusing ATPase	Involved in vesicle-mediated transport. Catalyzes the vesicles’ fusion with the Golgi cisternae. Acts as a fusion protein essential in the delivery of cargo proteins to the Golgi stack.	- Cytoplasm
**Periodontitis**			
THSD4	Thrombospondin type-1 domain-containing protein 4	Promotes the assembly of a FBN1 matrix. Attenuates TGFB signaling.	- Extracellular Matrix
NLGN1	Neuroligin-1	Cell surface protein involved in synapses and synaptic signal transmission, and recruits and clusters other synaptic proteins.	- Extracellular Region - Plasma membrane - Post-synaptic density
ACTN1	α-actinin-1	Bundling protein of F-actin that anchors actin intracellularly.	- Plasma membrane - Cytoskeleton
ACTN2	α-actinin-2	Bundling protein of F-actin that anchors actin intracellularly.	- Z line
ABCA1	ATP-binding cassette sub-family A member 1	Anion transporter dependent on cAMP sensitive to sulfonylurea.	- Endosome - Plasma Membrane - Membrane
SMURF2	E3 ubiquitin–protein ligase SMURF2	Involved in the transfer of the ubiquitin to targeted substrates. Interacts with SMAD1 and SMAD7 triggering ubiquitination and degradation.	- Plasma Membrane - Nucleus - Cytoplasm - Membrane Raft
IGF2R	Cation-independent mannose-6-phosphate receptor	Involved in the transport of phosphorylated lysosomal enzymes to lysosomes.	- Lysosome
HLA-DOA	HLA class II histocompatibility antigen, DO α chain	Modulates the HLA class II restricted antigen displaying path via the interaction with B-cells’ HLA-DM. Alters the peptide interchange activity of HLA-DM	- Lysosome - Endosome
**Parkinson’s Disease and Periodontitis**			
DLG2	Disks large homolog 2	Acts in chronic pain perception via NMDA receptor signaling. Regulates the stability of cholinergic synapses.	- Plasma membrane Other locations: - Postsynaptic density - Synapse - Axon - Perikaryon

## Supplementary Materials

The following are available online at https://www.mdpi.com/2073-4425/11/11/1385/s1, Table S1: Genes expressing protein variants related to Periodontitis, Table S2: Genes expressing protein variants related to Parkinson’s Disease, Table S3: Reactome pathways analysis results.
