# Supplementary material for: Network Protein Interaction in Parkinson’s Disease and Periodontitis Interplay: A Preliminary Bioinformatic Analysis
Source: Genes (Basel). 2020 Nov 23;11(11):1385. doi: 10.3390/genes11111385 (PMC7700320; doi:10.3390/genes11111385)
Supplement: Supplementary file 1 [file genes-11-01385-s001.pdf]

Table S1. Genes expressing protein variants related to Periodontitis

Mapped gene	Reported trait
GLT6D1	Periodontitis
AL360006.1, KCNK1	Periodontal microbiota
PKN2-AS1	Periodontal microbiota
CLIC5, RUNX2	Periodontal microbiota
Y_RNA, AC064802.1	Periodontal microbiota
CAMTA1	Periodontal microbiota
AF111167.2, LINC01220	Periodontal microbiota
TENM2	Periodontal microbiota
GRID1	Periodontal microbiota
AL360006.1, KCNK1	Periodontal microbiota
AC016751.2, EXTL2P1	Periodontal microbiota
DAB2IP, AL596244.1	Periodontal microbiota
Y_RNA, NAMPTP1	Periodontal microbiota
FAM166C, OTOF	Periodontal microbiota
FBXO38	Periodontal microbiota
UHRF2	Periodontal microbiota
TBC1D1	Periodontal microbiota
AC003044.1	Periodontitis
AL136967.2, FOXP4-AS1	Periodontitis
ADGRE1	Periodontitis
TTC6	Periodontitis (CDC/AAP)
GPN1	Periodontitis (CDC/AAP)
HLA-DOA	Periodontitis (CDC/AAP)
ITGA8	Periodontitis (CDC/AAP)
ERGIC1	Periodontitis (CDC/AAP)
LRP1B	Periodontitis (CDC/AAP)
PRB2, AC078950.1	Periodontitis (CDC/AAP)
C10orf91, AL451069.1	Periodontitis (CDC/AAP)
AL161449.2, Y_RNA	Periodontitis (CDC/AAP)
CEP295NL, TIMP2	Periodontitis (CDC/AAP)
ACTN2	Periodontitis (CDC/AAP)
KDM4B	Periodontitis (CDC/AAP)
LINC01828, AC007403.1	Periodontitis (CDC/AAP)
AC022239.2, LINC00208	Periodontitis (CDC/AAP)
AC015987.1	Periodontitis (CDC/AAP)
MFSD1	Periodontitis (CDC/AAP)
LINC01262, HSP90AA4P	Periodontitis (CDC/AAP)
IGF2R	Periodontitis (CDC/AAP)
WDR73, SCAND2P	Periodontitis (CDC/AAP)
PARP15	Periodontitis (CDC/AAP)
RPL35AP19, AC068413.1	Periodontitis (CDC/AAP)

CSMD1	Periodontitis (CDC/AAP)
ETNK2	Periodontitis (CDC/AAP)
AC010468.1, AC010468.3	Periodontitis (PAL4Q3)
THSD4	Periodontitis (PAL4Q3)
ROBO2	Periodontitis (PAL4Q3)
LINC01748, LINC02778	Periodontitis (PAL4Q3)
DEFA10P, DEFA9P	Periodontitis (PAL4Q3)
NKAIN3	Periodontitis (PAL4Q3)
PPIAP65, LINC01854	Periodontitis (PAL4Q3)
IGLV10-54	Periodontitis (PAL4Q3)
ROCK1P1	Periodontitis (Mean PAL)
AC055874.1	Periodontitis (Mean PAL)
SMURF2	Periodontitis (Mean PAL)
AC027229.1, RN7SL97P	Periodontitis (Mean PAL)
AC015871.1, ST20-MTHFS	Periodontitis (Mean PAL)
THSD4	Periodontitis (Mean PAL)
ZNF579	Periodontitis (Mean PAL)
ABCA1	Periodontitis (Mean PAL)
MIR8052, BAK1P2	Periodontitis (Mean PAL)
BCORL1, AL034405.1	Periodontitis (Mean PAL)
BIRC5	Periodontitis (Mean PAL)
AC063949.1, C12orf74	Periodontitis (Mean PAL)
ST20-AS1, AC015871.2	Periodontitis (Mean PAL)
CDH13, AC009063.2	Periodontitis (Mean PAL)
AC114324.2, LINC02062	Periodontitis (Mean PAL)
ICE2P2, NDUFA5P5	Periodontitis (Mean PAL)
AP001042.1	Periodontitis (Mean PAL)
AC093534.2, RPL35AP15	Periodontitis (DPAL)
DAOA-AS1, AL138954.1	Periodontitis (DPAL)
GRIK1	Periodontitis (DPAL)
AC093534.2, RPL35AP15	Periodontitis (DPAL)
NLGN1	Periodontitis (DPAL)
AC006059.2, CCDC13	Periodontitis (DPAL)
PWRN1, AC090983.2	Periodontitis (DPAL)
SUMF1	Periodontitis (DPAL)
LBP	Periodontitis (DPAL)
LINC02022	Periodontitis (DPAL)
AC005208.1, KCNJ16	Periodontitis (DPAL)
SCN2A	Periodontitis (DPAL)
TGIF1	Periodontitis (DPAL)
NPAP1, AC090983.2, PWRN1	Periodontitis (DPAL)
ZFPM2	Periodontitis (DPAL)
PPIAP65, LINC01854	Periodontitis (PAL4Q3)

LINC00907	Periodontitis (PAL4Q3)
RBFOX1	Periodontitis (PAL4Q3)
LINC01933	Periodontitis (PAL4Q3)
HMGB1P5, AC092421.1	Periodontitis (PAL4Q3)
DEFA10P, DEFA9P	Periodontitis (PAL4Q3)
LINC00907	Periodontitis (PAL4Q3)
THSD4	Periodontitis (PAL4Q3)
FAM135B	Periodontitis (PAL4Q3)
AC010468.1, CAMK4	Periodontitis (PAL4Q3)
FAM126A	Periodontitis (Mean PAL)
NKAIN2	Periodontitis (Mean PAL)
PSMA8	Periodontitis (Mean PAL)
ERC2	Periodontitis (Mean PAL)
MIR8052, BAK1P2	Periodontitis (Mean PAL)
ACTN1	Periodontitis (Mean PAL)
DKK1, RPL31P44	Periodontitis (Mean PAL)
AL591501.1	Periodontitis (Mean PAL)
FIZ1	Periodontitis (Mean PAL)
HMX3, ACADSB	Periodontitis (Mean PAL)
HS6ST2	Periodontitis (Mean PAL)
LINC01278	Periodontitis (Mean PAL)
AC090666.1, LINC01919	Periodontitis (Mean PAL)
NKAIN2	Periodontitis (Mean PAL)
HNRNPA1P58, NRSN1	Periodontitis (Mean PAL)
AC123023.1, LINC01811	Periodontitis (Mean PAL)
GDF15	Periodontitis (Mean PAL)
NPM1P2	Periodontitis (Mean PAL)
AC063965.1, PTEN	Periodontitis (Mean PAL)
PTPRT	Periodontitis (Mean PAL)
CSMD1	Periodontitis (CDC/AAP)
RGMA, AC108457.1	Periodontitis (CDC/AAP)
AC022239.2, LINC00208	Periodontitis (CDC/AAP)
AC022239.2, LINC00208	Periodontitis (CDC/AAP)
KDM4B	Periodontitis (CDC/AAP)
AC090365.1, CDH2	Periodontal disease-related phenotype (Socransky)
FHOD3	Periodontal disease-related phenotype (Socransky)
LINC02855, HAS2-AS1	Periodontal disease-related phenotype (Socransky)
HSP90AB2P, U6	Periodontal disease-related phenotype (Socransky)
GVINP1	Periodontal disease-related phenotype (Socransky)
AL356124.1	Periodontal disease-related phenotype (Socransky)
HSP90AB2P, U6	Periodontal disease-related phenotype (Socransky)
OSBPL10	Periodontal disease-related phenotype (Socransky)
AL355838.1	Periodontal disease-related phenotype (Socransky)

CDKL1	Periodontal disease-related phenotype (Socransky)
CRACR2A	Chronic periodontitis (localised)
KCNQ5	Periodontitis
GPR141, EPDR1	Periodontitis
C5AR1	Chronic periodontitis
DLG2	Chronic periodontitis
SIGLEC5, AC018755.2	Periodontitis
AP000959.1, MAPK6P2	Periodontitis
RNU6-675P, TEX51	Periodontitis
MIR297, LYPLA1P2	Periodontitis
AL109933.3	Periodontitis
LINC01239, AL391117.1	Periodontitis
AL354916.1, CUX2P1	Periodontitis
NUDT5	Periodontitis
AC004241.1	Periodontitis
HUNK	Periodontitis

Table S2. Genes expressing protein variants related to Parkinson’s Disease

Mapped gene	Reported trait
SIRLNT, AC022733.2	Parkinson's disease (motor and cognition)
LMNB1, MARCH3	Parkinson's disease (motor and cognition)
CLRN3	Parkinson's disease (motor and cognition)
ODAPH	Parkinson's disease (motor and cognition)
CTC1	Parkinson's disease (motor and cognition)
PLPPR1	Parkinson's disease (age at diagnosis)
AC093866.1	Parkinson's disease
LINC02210-CRHR1, LINC02210	Parkinson's disease
HLA-DQA1, HLA-DRB1	Parkinson's disease
RN7SL813P, STK39	Parkinson's disease
GAK	Parkinson's disease
GCH1	Parkinson's disease
MCCC1, DCUN1D1	Parkinson's disease
KLHL7-DT, FAM126A	Parkinson's disease
RAB29, NUCKS1	Parkinson's disease
TIAL1, RAD1P1	Parkinson's disease
FAM47E, FAM47E-STBD1, AC034139.1	Parkinson's disease
BST1	Parkinson's disease
RIT2	Parkinson's disease
AL009179.1	Parkinson's disease
CCDC62	Parkinson's disease
OR5AZ1P, OR5BD1P	Parkinson's disease
LINC02451	Parkinson's disease
SH3GL2	Parkinson's disease
SYT17	Parkinson's disease
NDUFAF2	Parkinson's disease
CA8	Parkinson's disease
AC067852.1	Parkinson's disease
LRRK2	Parkinson's disease
AC087311.2, SYT10	Parkinson's disease
GBA	Parkinson's disease
TMEM175	Parkinson's disease
BST1	Parkinson's disease
LRRK2	Parkinson's disease
LAMTOR2, RAB25	Parkinson's disease
CCNT2-AS1	Parkinson's disease
MCCC1	Parkinson's disease
AC093866.1	Parkinson's disease
RN7SL813P, STK39	Parkinson's disease
CCDC62	Parkinson's disease

AC093866.1	Parkinson's disease
GAK	Parkinson's disease
NSF	Parkinson's disease
SEMA5A	Parkinson's disease
TMEM72-AS1, AL356157.1	Parkinson's disease
STAP1	Parkinson's disease
DLG2	Parkinson's disease
AC093866.1	Parkinson's disease
BST1	Parkinson's disease
SLC2A13	Parkinson's disease
AC119673.1	Parkinson's disease
GAK	Parkinson's disease (familial)
PLEKHM1	Parkinson's disease
TAS1R2, PAX7	Parkinson's disease
BRINP1	Parkinson's disease
DGKQ	Parkinson's disease
AC034213.1, GFPT2	Parkinson's disease
SNCA	Parkinson's disease
FAM47E, FAM47E-STBD1, AC034139.1	Parkinson's disease
AC093866.1	Parkinson's disease
MAPT-AS1, SPPL2C	Parkinson's disease
MCCC1	Parkinson's disease
TMEM175	Parkinson's disease
RIT2	Parkinson's disease
SREBF1	Parkinson's disease
LRRK2	Parkinson's disease
SLC41A1	Parkinson's disease
AJ009632.2	Parkinson's disease
NSF	Parkinson's disease
UNC13B	Parkinson's disease
WNT3	Parkinson's disease
LINC01709, LINC01307	Parkinson's disease
AC093866.1	Parkinson's disease
AC093866.1	Parkinson's disease
HLA-DRA	Parkinson's disease
HLA-DRA	Parkinson's disease
WNT3	Parkinson's disease
WNT3	Parkinson's disease
LINC01709, LINC01307	Parkinson's disease
CCDC82	Parkinson's disease
TMC3-AS1, TMC3	Parkinson's disease
COL13A1	Parkinson's disease
LINC00476	Parkinson's disease (pesticide exposure interaction)

AC011586.2	Parkinson's disease
SPTSSB	Parkinson's disease
DLG2	Parkinson's disease
ZNF165, ZSCAN16-AS1	Parkinson's disease
LRRK2, AC079630.1	Parkinson's disease
CCDC62	Parkinson's disease
GCH1	Parkinson's disease
TMEM229B	Parkinson's disease
AC018618.1	Parkinson's disease
BCKDK	Parkinson's disease
MAPT	Parkinson's disease
RIT2	Parkinson's disease
TMPRSS9	Parkinson's disease
LZTS3, DDRGK1	Parkinson's disease
ITPKB	Parkinson's disease
MAP4K4	Parkinson's disease
SCN2A	Parkinson's disease
TBC1D5	Parkinson's disease
IP6K2	Parkinson's disease
ITIH1	Parkinson's disease
RNU1-138P, CAMK2D	Parkinson's disease
NDUFAF2	Parkinson's disease
GPR89P, AL009179.1	Parkinson's disease
CTSB	Parkinson's disease
HLA-DQB1, MTCO3P1	Parkinson's disease
GPNMB	Parkinson's disease
AGAP1	Parkinson's disease
INPP5F	Parkinson's disease
PAM	Parkinson's disease
AP001979.1, IGSF9B	Parkinson's disease
CCN6, LINC02527	Parkinson's disease
AC103957.1, AC103957.2	Parkinson's disease
GBF1	Parkinson's disease
CAB39L	Parkinson's disease
AL359502.1, PSMA6P4	Parkinson's disease
AL355773.1, KTN1	Parkinson's disease
LTK	Parkinson's disease
ITGA2B	Parkinson's disease
Y_RNA, MED13	Parkinson's disease
HMGN2P18, KRTCAP2	Parkinson's disease
RAB29, NUCKS1	Parkinson's disease
SIPA1L2	Parkinson's disease
CCNT2-AS1	Parkinson's disease

RN7SL813P, STK39	Parkinson's disease
MCCC1	Parkinson's disease
TMEM175	Parkinson's disease
BST1	Parkinson's disease
FAM47E, FAM47E-STBD1, AC034139.1	Parkinson's disease
AC093866.1	Parkinson's disease
BIN3	Parkinson's disease
SH3GL2	Parkinson's disease
ITGA8	Parkinson's disease
GPR65	Parkinson's disease
SYT17	Parkinson's disease
CASC16	Parkinson's disease
WNT9A, CICP26	Parkinson's disease
COL5A2	Parkinson's disease
AC126121.3	Parkinson's disease
AC068633.1, IGSF11	Parkinson's disease
LINC02224	Parkinson's disease
AL513124.1, AL355674.1	Parkinson's disease
RPA2P1, MDGA2	Parkinson's disease
AC007998.4, AC007998.3	Parkinson's disease
LHFPL2	Parkinson's disease (familial, age at onset)
TRPS1	Parkinson's disease (familial, age at onset)
KLHDC1	Parkinson's disease (familial, age at onset)
TPM1	Parkinson's disease (familial, age at onset)
LINC02210, LINC02210-CRHR1	Parkinson's disease
AC093866.1	Parkinson's disease
BST1	Parkinson's disease
AC079385.1	Parkinson's disease
HLA-DRA	Parkinson's disease
PRDM15	Parkinson's disease
AC093866.1	Parkinson's disease
RN7SL813P, STK39	Parkinson's disease
BST1	Parkinson's disease
AL357075.5, CNKSR3	Parkinson's disease
TSBP1-AS1, HLA-DRA	Parkinson's disease
SLC50A1	Parkinson's disease
CCNT2-AS1	Parkinson's disease
DGKQ	Parkinson's disease
CTSB, FDFT1	Parkinson's disease
SH3GL2	Parkinson's disease
ANO5, AC116534.1	Parkinson's disease
SLC2A13	Parkinson's disease
AC018618.1	Parkinson's disease

PRSS53, AC135050.2, ZNF646	Parkinson's disease
WNT3	Parkinson's disease
RIT2	Parkinson's disease
DSG3	Parkinson's disease (age of onset)
AC021979.2, OCA2	Parkinson's disease (age of onset)
ATF6	Parkinson's disease (age of onset)
QSER1, PRRG4	Parkinson's disease (age of onset)
AAK1	Parkinson's disease (age of onset)
CNTN1	Parkinson's disease
KANSL1	Parkinson's disease
KCNN3, PMVK	Parkinson's disease
RAB29, NUCKS1	Parkinson's disease
MCCC1	Parkinson's disease
AC093866.1	Parkinson's disease
SLC2A13	Parkinson's disease
DLG2	Parkinson's disease
COL3A1, AC092598.1	Parkinson disease and lewy body pathology
TCEANC2	Parkinson disease and lewy body pathology
MX2	Parkinson disease and lewy body pathology
ZP3	Parkinson disease and lewy body pathology
PABPN1L, TRAPPC2L	Parkinson disease and lewy body pathology
KCNIP4	Parkinson disease and lewy body pathology
FRG1CP, MIR663AHG	Parkinson disease and lewy body pathology
HTR2A-AS1, HTR2A	Parkinson disease and lewy body pathology
AC026826.3, AC023968.1	Parkinson disease and lewy body pathology
AC079070.1, LINC02582	Parkinson disease and lewy body pathology
ISM1, AL121782.1	Parkinson's disease
FAM47E, FAM47E-STBD1, AC034139.1	Parkinson's disease
ITGA8	Parkinson's disease
SNCA	Parkinson's disease
NSF	Parkinson's disease
LINC02210-CRHR1, LINC02210	Parkinson's disease
NUCKS1	Parkinson's disease
CYP17A1, WBP1L	Parkinson's disease
MMRN1, SNCA-AS1	Parkinson's disease
AL365295.1, LINC02331	Parkinson's disease
PMVK	Parkinson's disease or first degree relation to individual with Parkinson's disease
FAM47E, SCARB2	Parkinson's disease or first degree relation to individual with Parkinson's disease
GBA	Parkinson's disease or first degree relation to individual with Parkinson's disease
AC093866.1	Parkinson's disease or first degree relation to individual with Parkinson's disease
Z98751.1, VAMP4	Parkinson's disease or first degree relation to individual with Parkinson's disease

RNU1-138P, CAMK2D	Parkinson's disease or first degree relation to individual with Parkinson's disease
RAB29	Parkinson's disease or first degree relation to individual with Parkinson's disease
ELOVL7	Parkinson's disease or first degree relation to individual with Parkinson's disease
SIPA1L2	Parkinson's disease or first degree relation to individual with Parkinson's disease
KCNS3	Parkinson's disease or first degree relation to individual with Parkinson's disease
RSL24D1P1, AL009179.2	Parkinson's disease or first degree relation to individual with Parkinson's disease
TRIM40	Parkinson's disease or first degree relation to individual with Parkinson's disease
HLA-DRB1, HLA-DQA1	Parkinson's disease or first degree relation to individual with Parkinson's disease
RNU4-66P, RIMS1	Parkinson's disease or first degree relation to individual with Parkinson's disease
CCN6, LINC02527	Parkinson's disease or first degree relation to individual with Parkinson's disease
AL137783.1, HMGB1P13	Parkinson's disease or first degree relation to individual with Parkinson's disease
GPNMB	Parkinson's disease or first degree relation to individual with Parkinson's disease
AC006001.3, AC008267.3	Parkinson's disease or first degree relation to individual with Parkinson's disease
CTSB	Parkinson's disease or first degree relation to individual with Parkinson's disease
AC011586.2	Parkinson's disease or first degree relation to individual with Parkinson's disease
BIN3	Parkinson's disease or first degree relation to individual with Parkinson's disease
FAM49B	Parkinson's disease or first degree relation to individual with Parkinson's disease
SH3GL2	Parkinson's disease or first degree relation to individual with Parkinson's disease
SH3GL2	Parkinson's disease or first degree relation to individual with Parkinson's disease
UBAP2	Parkinson's disease or first degree relation to individual with Parkinson's disease
CHRNB1	Parkinson's disease or first degree relation to individual with Parkinson's disease
RETREG3	Parkinson's disease or first degree relation to individual with Parkinson's disease
UBTF	Parkinson's disease or first degree relation to individual with Parkinson's disease
FAM171A2	Parkinson's disease or first degree relation to individual with Parkinson's disease
LINC02210-CRHR1	Parkinson's disease or first degree relation to individual with Parkinson's disease
LINC02210-CRHR1	Parkinson's disease or first degree relation to individual with Parkinson's disease
WNT3	Parkinson's disease or first degree relation to individual with Parkinson's disease
BRIP1	Parkinson's disease or first degree relation to individual with Parkinson's disease
DNAH17	Parkinson's disease or first degree relation to individual with Parkinson's disease
ASXL3	Parkinson's disease or first degree relation to individual with Parkinson's disease

RIT2	Parkinson's disease or first degree relation to individual with Parkinson's disease
SRSF10P1, SMAD4	Parkinson's disease or first degree relation to individual with Parkinson's disease
SPPL2B, AC005258.1	Parkinson's disease or first degree relation to individual with Parkinson's disease
AL035461.3, CRLS1	Parkinson's disease or first degree relation to individual with Parkinson's disease
DYRK1A	Parkinson's disease or first degree relation to individual with Parkinson's disease
AL135927.1, SEMA4A	Parkinson's disease or first degree relation to individual with Parkinson's disease
TMEM163	Parkinson's disease or first degree relation to individual with Parkinson's disease
AC093866.1	Parkinson's disease or first degree relation to individual with Parkinson's disease
SNCA	Parkinson's disease or first degree relation to individual with Parkinson's disease
ZNF608, AC113398.1	Parkinson's disease or first degree relation to individual with Parkinson's disease
SLC44A4, EHMT2-AS1	Parkinson's disease or first degree relation to individual with Parkinson's disease
DNM1L, FGD4	Parkinson's disease or first degree relation to individual with Parkinson's disease
MUC19	Parkinson's disease or first degree relation to individual with Parkinson's disease
MUC19	Parkinson's disease or first degree relation to individual with Parkinson's disease
AC090630.1	Parkinson's disease or first degree relation to individual with Parkinson's disease
SCARB2, AC110795.1	Parkinson's disease or first degree relation to individual with Parkinson's disease
HMGN2P18, KRTCAP2	Parkinson's disease or first degree relation to individual with Parkinson's disease
FAM47E-STBD1, AC034139.1, FAM47E	Parkinson's disease or first degree relation to individual with Parkinson's disease
AL592295.3, FCGR2A	Parkinson's disease or first degree relation to individual with Parkinson's disease
AC093866.1	Parkinson's disease or first degree relation to individual with Parkinson's disease
RAB29, NUCKS1	Parkinson's disease or first degree relation to individual with Parkinson's disease
CLCN3	Parkinson's disease or first degree relation to individual with Parkinson's disease
ITPKB	Parkinson's disease or first degree relation to individual with Parkinson's disease
PAM	Parkinson's disease or first degree relation to individual with Parkinson's disease
AC006077.1	Parkinson's disease or first degree relation to individual with Parkinson's disease
KCNIP3	Parkinson's disease or first degree relation to individual with Parkinson's disease
MAP4K4	Parkinson's disease or first degree relation to individual with Parkinson's disease
TMEM163	Parkinson's disease or first degree relation to individual with Parkinson's disease
RN7SL813P, STK39	Parkinson's disease or first degree relation to individual with Parkinson's disease
TBC1D5	Parkinson's disease or first degree relation to individual with Parkinson's disease

RBMS3, AC098650.1	Parkinson's disease or first degree relation to individual with Parkinson's disease
IP6K2	Parkinson's disease or first degree relation to individual with Parkinson's disease
KPNA1	Parkinson's disease or first degree relation to individual with Parkinson's disease
MED12L	Parkinson's disease or first degree relation to individual with Parkinson's disease
SPTSSB	Parkinson's disease or first degree relation to individual with Parkinson's disease
MCCC1	Parkinson's disease or first degree relation to individual with Parkinson's disease
GAK	Parkinson's disease or first degree relation to individual with Parkinson's disease
TMEM175	Parkinson's disease or first degree relation to individual with Parkinson's disease
BST1	Parkinson's disease or first degree relation to individual with Parkinson's disease
LCORL	Parkinson's disease or first degree relation to individual with Parkinson's disease
ITGA8	Parkinson's disease or first degree relation to individual with Parkinson's disease
GBF1	Parkinson's disease or first degree relation to individual with Parkinson's disease
BAG3	Parkinson's disease or first degree relation to individual with Parkinson's disease
INPP5F	Parkinson's disease or first degree relation to individual with Parkinson's disease
RNF141	Parkinson's disease or first degree relation to individual with Parkinson's disease
DLG2	Parkinson's disease or first degree relation to individual with Parkinson's disease
IGSF9B	Parkinson's disease or first degree relation to individual with Parkinson's disease
LRRK2, AC079630.1	Parkinson's disease or first degree relation to individual with Parkinson's disease
LRRK2	Parkinson's disease or first degree relation to individual with Parkinson's disease
AC084878.1, SLC38A1	Parkinson's disease or first degree relation to individual with Parkinson's disease
HIP1R	Parkinson's disease or first degree relation to individual with Parkinson's disease
FBRSL1, AC079031.2	Parkinson's disease or first degree relation to individual with Parkinson's disease
CAB39L	Parkinson's disease or first degree relation to individual with Parkinson's disease
LINC00456, MBNL2	Parkinson's disease or first degree relation to individual with Parkinson's disease
MIPOL1	Parkinson's disease or first degree relation to individual with Parkinson's disease
GCH1	Parkinson's disease or first degree relation to individual with Parkinson's disease
RPS6KL1	Parkinson's disease or first degree relation to individual with Parkinson's disease
RNU6-835P, GPR65	Parkinson's disease or first degree relation to individual with Parkinson's disease
AC018618.1	Parkinson's disease or first degree relation to individual with Parkinson's disease
SYT17	Parkinson's disease or first degree relation to individual with Parkinson's disease

RABEP2, CD19	Parkinson's disease or first degree relation to individual with Parkinson's disease
SETD1A	Parkinson's disease or first degree relation to individual with Parkinson's disease
NOD2	Parkinson's disease or first degree relation to individual with Parkinson's disease
CASC16	Parkinson's disease or first degree relation to individual with Parkinson's disease
PHBP21	Parkinson's disease or first degree relation to individual with Parkinson's disease
GXYLT1	Parkinson's disease or first degree relation to individual with Parkinson's disease
AC003070.7, AC003070.2	Parkinson's disease or first degree relation to individual with Parkinson's disease
LINC02210-CRHR1	Parkinson's disease or first degree relation to individual with Parkinson's disease
MAPT-AS1	Parkinson's disease or first degree relation to individual with Parkinson's disease
KANSL1	Parkinson's disease or first degree relation to individual with Parkinson's disease
NSF	Parkinson's disease or first degree relation to individual with Parkinson's disease
LZTS3, DDRGK1	Parkinson's disease or first degree relation to individual with Parkinson's disease
AC093866.1	Parkinson's disease in GBA mutation carriers
CTSB	Parkinson's disease in GBA mutation carriers
MAPT	Parkinson's disease
RBMS3, AC098650.1	Parkinson's disease
AC093866.1	Parkinson's disease
LRRK2	Parkinson's disease
LRRK2	Parkinson's disease
DGKQ	Parkinson's disease
AC093866.1	Parkinson's disease
MMRN1, SNCA-AS1	Parkinson's disease
SNCA	Parkinson's disease (age of onset)
SNCA-AS1	Parkinson's disease (age of onset)
TMEM175	Parkinson's disease (age of onset)
APOE	Parkinson's disease (age of onset)
GCH1	Parkinson's disease
TMEM229B	Parkinson's disease
AC018618.1	Parkinson's disease
BCKDK	Parkinson's disease
MAPT	Parkinson's disease
RIT2	Parkinson's disease
TMPRSS9	Parkinson's disease
LZTS3, DDRGK1	Parkinson's disease
AC011586.2	Parkinson's disease
HMGN2P18, KRTCAP2	Parkinson's disease
RAB29, NUCKS1	Parkinson's disease
SIPA1L2	Parkinson's disease

CCNT2-AS1	Parkinson's disease
RN7SL813P, STK39	Parkinson's disease
MCCC1	Parkinson's disease
TMEM175	Parkinson's disease
BST1	Parkinson's disease
FAM47E, FAM47E-STBD1, AC034139.1	Parkinson's disease
AC093866.1	Parkinson's disease
HLA-DQB1, MTCO3P1	Parkinson's disease
GPNMB	Parkinson's disease
INPP5F	Parkinson's disease
DLG2	Parkinson's disease
AP001979.1, IGSF9B	Parkinson's disease
LRRK2, AC079630.1	Parkinson's disease
CCDC62	Parkinson's disease
SNCA	Parkinson's disease (age of onset)
PRKN	Parkinson's disease (age of onset)
AC093866.1	Parkinson's disease
LRRK2, MUC19	Parkinson's disease
KANSL1	Parkinson's disease
HLA-DQB1, MTCO3P1	Parkinson's disease
LINC02210-CRHR1	Parkinson's disease
TMEM175	Parkinson's disease
SETD1A	Parkinson's disease
CHL1-AS1, CHL1	Parkinsonism in frontotemporal lobe dementia
SP1	Parkinsonism in frontotemporal lobe dementia
LINC01500	Parkinsonism in frontotemporal lobe dementia

Table S3. Reactome pathways analysis results

Reactome pathways	Number of genes	Number of genes uploaded in the PPI	expected	Fold Enrichment	+/-	Fisher Test	False Discovery Rate
Plasma lipoprotein assembly	18	2	.02	> 100	+	1.32E-04	3.78E-02
Ras activation upon Ca2+ influx through NMDA receptor	19	2	.02	> 100	+	1.46E-04	3.71E-02
↳CREB1 phosphorylation through NMDA receptor-mediated activation of RAS signaling	27	2	.02	85.81	+	2.81E-04	4.02E-02
Negative regulation of NMDA receptor-mediated neuronal transmission	21	2	.02	> 100	+	1.76E-04	4.02E-02
Unblocking of NMDA receptors, glutamate binding and activation	21	2	.02	> 100	+	1.76E-04	3.65E-02
Long-term potentiation	22	2	.02	> 100	+	1.92E-04	3.37E-02
Nephrin family interactions	23	2	.02	> 100	+	2.08E-04	3.17E-02
Golgi Associated Vesicle Biogenesis	54	4	.05	85.81	+	1.60E-07	1.82E-04
↳trans-Golgi Network Vesicle Budding	72	4	.06	64.35	+	4.78E-07	3.64E-04
↳Membrane Trafficking	625	6	.54	11.12	+	1.02E-05	3.87E-03
Cargo recognition for clathrin-mediated endocytosis	104	3	.09	33.42	+	1.01E-04	3.30E-02
↳Clathrin-mediated endocytosis	144	5	.12	40.22	+	1.38E-07	3.15E-04
Platelet degranulation	127	3	.11	27.36	+	1.80E-04	3.43E-02
↳Response to elevated platelet cytosolic Ca2+	132	3	.11	26.33	+	2.01E-04	3.28E-02
Unclassified	10195	0	8.80	< 0.01	-	8.19E-06	3.74E-03
